# Analysis of variant interactions in families with autism points to genes involved in the development of the central nervous system

**DOI:** 10.1371/journal.pone.0326022

**Published:** 2025-06-11

**Authors:** Jacek Kaczyński, Marta Pasenkiewicz-Gierula

**Affiliations:** Department of Biochemistry, Biophysics and Biotechnology, Jagiellonian University in Kraków, Kraków, Poland; Shiga Medical Center, JAPAN

## Abstract

Whole-genome sequencing data of simplex families with autism spectrum disorder (ASD) were analyzed by searching for statistical interactions between loci. The resulting variant pairs mapped to 411 genes, of which 368 had not been associated with ASD before. The variants were used to build an ASD predictor based on an open-source machine learning library. The predictor correctly classifies over 78% of samples from a test set with an average significance level of 8.9· 10^-158^. Gene Ontology (GO) enrichment analysis of the identified risk genes points to functions related to the development of the Central Nervous System (CNS). Clustering cases on the basis of risk variants improves predictor accuracy and reveals additional overrepresented GO terms. Some of the detected statistical interactions can be linked to known biological interactions between genes involved in the development of the CNS. Analysis of the statistical interactions also points to genes whose biological functions are not yet known.

## Introduction

Autism spectrum disorder (ASD) is characterized by deficits in social interaction and communication, as well as repetitive and restrictive behaviors, with its onset in early development. Studies of families and twins show that ASD is highly heritable [1,2]. For example, later-born siblings of children with ASD are 30 times more likely to develop ASD compared to the siblings of those without a diagnosis [3]. An epidemiological study from Sweden estimates the genetic contribution to ASD liability at 59% [4]. Majority of the genetic risk comes from additive effect of common inherited variants (49%). The contribution of the *de novo* variants is estimated at 3%. Less than 10% of ASD cases have been linked to rare protein disrupting mutations and roughly half of these are associated with syndromic autism where ASD is comorbid with a rare genetic disease [5]. However, genome-wide association studies (GWAS) have identified few common inherited variants responsible for ASD that are statistically significant [6]. The risk variants that are statistically significant usually account for syndromic ASD and explain a small percentage of cases [5]. As a result, most cases of idiopathic ASD cannot be linked to specific genetic variants [6]. Some authors attribute this fact to insufficient sample size [6]. However, common inherited risk variants must also be carried by some unaffected family members which means they are weakly correlated with ASD. This limitation is unlikely to be addressed by increasing the sample size. An effective search for inherited genetic variants responsible for ASD requires the use of new statistical methods. The main goal of this study is to propose such a method and apply it to identify genetic variants responsible for idiopathic ASD, both inherited and *de novo*.

As autism is believed to be polygenic [5], one can expect statistical interactions between multiple loci contributing to the condition. In practice, it means that a pair of variants meets the assumed significance level threshold, while each of the variants is not significant when evaluated individually.

Since the number of unique variant pairs exceeds 10^18^, checking all of them is prohibitively time consuming. While evaluating all possible combinations of two or more loci is not computationally feasible, approximate methods do exist. Here we apply the two stage approach proposed by *Marchini et al* [7]. Initially, all variants weakly associated with the condition are preselected by applying an initial screening significance level p_i_. Next all pairs of the preselected variants are evaluated by applying a target significance level p_t_ which takes into account appropriate correction for multiple testing.

The interacting pairs of variants are used to build an ASD predictor to classify the members of the cohort on the basis of the genetic data. The predictor is instrumental in understanding the impact of the findings in terms of percentages of cases and controls which can be explained by the interacting variants. Additionally, the predictor can be used to evaluate the effectiveness of the proposed method of identifying risk variants by comparing its significance level with similar predictors developed based on GWAS.

The resulting pairs of variants are further studied by applying GO enrichment analysis. While some of the variants are located in intergenic areas, many of them are located within genes. Converting pairs of variants to pairs of genes leads to a network of genes which can be assessed for GO term overrepresentation. The analysis may be instrumental in understating the nature of genetic changes contributing to the ASD risk. It is expected that the results of GO analysis can be mapped to known characteristics of ASD, which provides additional arguments for the effectiveness of the applied approach.

Because ASD is diagnosed solely on the basis of symptoms [5], it may be a mix of unrelated genetic conditions. This hypothesis can be tested by clustering cases based on identified risk variants and analyzing the resulting clusters for GO overrepresentation. A prerequisite for the clustering is the ability to explain most of the cases from genetic data. This ambitious goal has not been achieved by any ASD research to date but is achieved by this study.

The statistical interactions detected between loci can be validated by mapping them to biological interactions between genes or their products. Such an analysis may also shed more light on the biological roles of the impacted genes. Since ASD largely involves unique human traits such as speech, it can be expected that some risk genes will also be human-specific. Therefore, the analysis of statistical gene interactions responsible for ASD may help identify genes responsible for the development and functioning of the human CNS, which are difficult to discover by other methods.

## Materials and methods

### Data preparation and variant pair search

This study leverages the Simons Foundation Autism Research Initiative (SFARI) whole-genome dataset [8], which contains genetic and phenotypic data for 1926 families consisting of an autistic child, an unaffected sibling, and parents (data retrieved 6/19/2020–11/23/2020). Genetic data consists of whole-genome sequence variations stored in standard VCF files. The files were preprocessed by removing reference variants. While one cannot completely rule out contributions from reference variants, they are less likely to be significant and without this additional restriction the pair search would have been prohibitively time consuming. This assumption is ultimately verified by the quality of case status predictors based on the identified risk variants, since removing reference variants can only cause false negatives. Additionally, allele depth (AD) was used as an input filter. Variants with a low allele depth are likely to be somatic mutations [9] that likely do not contribute to the genetic condition with onset in early childhood or sequencing errors. The remaining variants were screened to detect variants weakly associated with ASD at the adopted initial screening level of 10^-3^. This value is a compromise between the accuracy of the search and the computational feasibility, since the duration of the second step of the pair search is proportional to the square of the number of the pre-selected variants. Variant significance levels used for the initial screening were based on the Chi Square test for the standard 2x2 contingency table representing occurrences of variants in cases and controls. The pre-selected variants were used to create variant pairs. The second step of the analysis identifies all pairs which meet the final significance level threshold of 1.3·10^-5^. The final significance level includes the Bonferroni correction for multiple tests. Significance levels of variant pairs are calculated based on occurrences of pairs in cases and controls. A variant pair is present if both variants are present. The input VCF files were processed using a custom Java application based on the HTSJDK open-source library (source code available at https://github.com/samtools/htsjdk).

The initial screening was performed for two minimum allele depth values, namely 0% and 25%. 0% is the default level where all variants present in the VCF file which passed read quality thresholds are taken into consideration. The 25% threshold means that a given variant is more likely to be present than absent if it is heterozygous. The results shown in Table 1 include a high number of variants which appear to be significantly associated with autism on the sex chromosomes. Many of these are false positives and an artifact of the gender imbalance given that 86.8% of cases are males compared to 47.4% of the controls. Such a high percentage of impacted males is typical of ASD [5]. While the number of false positives could be reduced by removing sex chromosomes from the analysis, such an approach is likely to eliminate risk variants responsible for the gender-specific ASD risk. Therefore, the following approaches for dealing with the gender imbalance were devised: a) Skip families with gender mismatch, and b) Replace mismatched siblings with one of the parents.

**Table 1:** Statistics of the initial variant search.

Minimum Allele Depth	chr1-chr22 p < 10^−3^	chrX, chrY p < 10^−3^	chr1-chr22 p < 1.3·10^-5^	chrX, chrY p < 1.3·10^-5^
0	10061	713683	2533	546858
25	5974	698772	1541	537656

Number of significant variants per minimum allele depth for two significance thresholds p_i_ = 10^-3^ and p_t_ = 1.3·10^-5^. Results are grouped by autosomes and sex chromosomes.

Applying each of the two approaches reduces the number of potential false positives on sex chromosomes from over half a million to single digit numbers, as shown in Table 2. Setting the minimum AD level at 25% was also helpful because it reduced the number of putative false positives on autosomes. The SFARI WGS cohort was prepared by removing cases where ASD was caused by known rare mutations [10] and hence the significant variants on autosomes are considered false positives too. Each false positive introduced in the initial screening phase is likely to cause many false positives in the final step and hence a conservative approach is better. While both methods were effective in dealing with gender imbalance, skipping families reduces the statistical power of the dataset and therefore henceforth the sibling replacement approach will be used.

**Table 2:** Statistics of variant search after applying gender mismatch corrections.

Approach to gender mismatch	Minimum Allele Depth	chr1-chr22 p < 10^−3^	chrX, chrY p < 10^−3^	chr1-chr22 p < 1.3·10^-5^	chrX, chrY p < 1.3·10^-5^
Skip family	0	3740	173	17	–
Skip family	25	2353	86	8	1
Replace sibling	0	6826	304	33	1
Replace sibling	25	4904	177	20	2

Number of significant variants per minimum allele depth for two significance threshold p_i_ = 10^-3^ and p_t_ = 1.3·10^-5^ for both approaches to gender mismatch. Results are grouped by autosomes and sex chromosomes.

### Analysis of the risk variants

The variant pairs generated by the previous steps were converted to a network of genes by mapping each intra-gene variant to the corresponding gene. Pairs containing inter-gene variants were skipped, as were pairs containing variants which belonged to pseudogenes and gene overlap areas. Pseudogenes were omitted because they have no biological function. The gene overlap areas were omitted because we could not find any statistically neutral way of identifying a single gene. Translating a variant pair to multiple gene pairs would also introduce a bias.

Since the variant pairs are significantly associated with the ASD, the corresponding genes can be considered ASD risk gene candidates. The Simons Foundation, which provided the dataset, also maintains the Gene Scoring Module in an effort to assess the strength of evidence associated with risk genes [9]. The novelty of the gene list obtained by the pair search can be assessed using the Scoring Module lookup. There are four scores 1 – High Confidence, 2 – Strong Candidate, 3- Suggestive Candidate, S – Syndromic. The “S” score can coexist with a numerical score, so they represent different dimensions. Lack of the SFARI score typically means a gene has not been previously associated with autism or the provided evidence was not conclusive versus the criteria established by the curators of the database [11].

In order to better understand the biological function of the identified risk genes, a GO term overrepresentation analysis was performed using the tool BiNGO [12]. The biological process aspect was selected as the basis for the annotations. The term overrepresentation was assessed using the hypergeometric test with a FDR correction and a minimum significance level of 0.05 after the correction. The whole annotation was selected as a reference set.

The variants identified via the pair search were used to build an ASD predictor based on the open-source library Apache Spark. The library contains several classifier models, which could be applied to predict the ASD from variants. The following classifiers were evaluated: Logistic Regression, Decision Tree, Random Forest, Gradient Boosted Tree, Multilayer Perceptron, Support Vector Machine, Naive Bayes and Factorization Machines. The best algorithm was selected by comparing accuracy and the Area Under ROC Curve (AUC) on the sample subsets set aside for the evaluation. In order to train a classifier, the cohort was split into a training set and a test set at a ratio of 67:33. The training and test data sets did not overlap to ensure unbiased assessment of the model fit. The splitting was accomplished by pseudo-random permutations of the families. The splitting, training and testing cycle was repeated 100 times. The machine learning models typically require the number of samples used to train the algorithm to be much higher than the number of model features. In order to improve the ratio, all family members were included in the training and test data sets. Since the resulting dataset is imbalanced in terms of case to control ratio, both classifier inputs and the metrics were weighted if possible. The weighting was applied to all classifiers except the Multilayer Perceptron (MP) and the Factorization Machines (FM) for which it was not supported by the Apache Spark library.

### Analysis of sample clusters

Since ASD is diagnosed purely on the basis of symptoms, it can be a mix of unrelated genetic diseases. In order to study this hypothesis, the set of samples, i.e., the cases and their family members is split into clusters. Clustering is accompanied by reducing the number of risk variants and risk genes using machine learning feature selection methods. The obtained reduced gene sets can be analyzed for GO overrepresentation and potential differences can be used to verify the hypothesis that we are dealing with multiple genetic diseases.

The clustering is often performed using the K-means algorithm or one of its derivatives [13]. K-means represents clusters by centroids calculated by averaging each feature for all members of a cluster. In this study, all features are binary, representing the presence of a given variant in a sample. Thus, centroids are difficult to interpret because of the fractional feature values. Partitioning Around Medoids (PAM) provides an alternative solution where clusters are represented by selected samples (medoids) [14], which are easier to interpret in the context of a genetic study. Both K-means and PAM require another method to determine the number of clusters in the dataset. Here we applied the gap statistics method which compares clustering accuracy with an appropriate random distribution [15]. Clustering can be applied to the entire dataset or to the cases only. The former means members of the same family can end up in different clusters, and the latter that they follow the corresponding case. The latter approach was found beneficial in terms of the accuracy of the resulting case status predictors. Clustering is followed by selecting variants most relevant for each cluster in order to understand differences in contributing genes and associated GO terms. It is also required in order to maintain an appropriate ratio of model features to observations and thus avoid predictor overtraining. Variants were selected by applying an Univariate Feature Selector (UFS), which retains variants on the basis of a predefined False Positive Rate (FPR) [16]. The UFS works by selecting the most relevant features of a dataset based on univariate statistical tests. Further information can be obtained from the Apache Spark website: https://spark.apache.org/docs/latest/ml-features.html#univariatefeatureselector. The FPR method selects features by comparing the Chi Square p-value with a predefined threshold. The FPR threshold was determined by repeating the clustering for a range of values and evaluating the performance of the ASD predictors built for each cluster based on the reduced variant set. The final FPR threshold maximizes the average accuracy of the per-cluster predictors. The resulting clusters are further investigated by applying Gene Ontology analysis to the associated subsets of variants.

### Approvals

The study was approved by the Bioethics Committee of the Jagiellonian University in Krakow, opinion No 1072.6120.2.2020.

## Results and discussion

### Results of pair search

**Fig 1:**
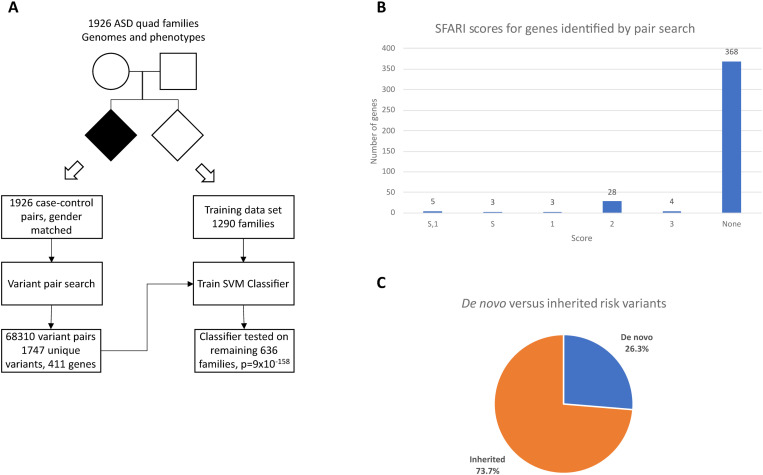
Results of the pair search. **(A)** Variant pair search produced 68,310 significant pairs containing 1,747 unique variants. An SVM classifier was trained on a set of 1,290 families and tested on the remaining 636 families with a p = 8.9·10^-158^. **(B)** SFARI scores of the 411 unique genes identified by the pair search. **(C)** Percentage of *de novo* versus inherited variants.

The pair search yielded 68,310 variant pairs meeting the assumed significance threshold, as shown in Fig 1A. Out of those, 23,005 variant pairs can be mapped to gene pairs skipping pseudogenes, gene overlap areas and inter-gene areas. The mapping of the variant pairs to pairs of genes generates 4,673 unique gene pairs, which contain 411 unique genes. The significant pairs contain 1,747 unique variants. The list of the gene pairs with the count of associated variant pairs is provided in the Supporting Information S1 Table. 4.6% of the risk variants were located in exons, 38.2% in non-coding regions of protein coding genes, 11.2% mapped to lncRNA genes, 1.8% to pseudogenes and the remaining 44.2% are located in intergenic regions.

Of the 411 genes, 368 have no SFARI score (Fig 1B), so we considered them novel risk genes in the context of autism genetics. The complete list of the risk genes with their SFARI scores and main functions, if known, is given in Supporting Information S2 Table. One of the objectives of this study was to take into account the contribution of inherited variants and we used the results of the pair search to determine how the goal would be met. Of all the occurrences of risk variants in the assessed families, 26.3% were *de novo* and the remaining 73.7% were inherited compared to 5% and 95% predicted by the epidemiological model [4], respectively (Fig 1C). While inherited variants dominate as expected, the percentage of *de novo* variants is significantly higher. The difference means that either the inherited variants carry more risk, or there is a difference between the populations analyzed. Part of the difference may be due to the method of estimating *de novo* liability in the epidemiological study which is based on analysis of copy number variation or loss-of-function mutations. The biological functions of 40–50% of the risk genes identified in this study are unknown and may therefore be omitted from loss-of-function analyses. It should be stressed that individual variants are typically *de novo* in one family and inherited in another (97%), which could be another reason for the difference.

### Predicting case status

The variants identified by the previous analyses were used to build classifiers. The Support Vector Machine (SVM) classifier outperformed all the others in terms of the AUC on the test datasets (Table 3). It also beat other classifiers with weighted inputs in terms of accuracy. On average, the model classifies 78.5% of samples from the test dataset correctly with a significance level of 8.9· 10^-158^ and an AUC of 0.87. The average significance level was calculated by averaging logarithms because the values are very small. The results of each training and testing cycle are provided in the Supporting Information S3 Table. A similar performance on the training and the test datasets indicates a good fit. The MP and the FM model achieved good accuracy at the expense of the AUC because of the lack of weighting.

**Table 3:** Performance of the evaluated classifiers on the training and the test data sets.

Classifier	Dataset	Accuracy %	Significance Level	AUC
Decision Tree	Training	61.7	2.6· 10^-52^	0.51
Decision Tree	Test	54.4	2.7· 10^−5^	0.52
Factorization Machines	Training	94.0	0.0	0.83
Factorization Machines	Test	82.1	5.2· 10^-145^	0.58
Gradient Boosted Tree	Training	84.9	0.0	0.92
Gradient Boosted Tree	Test	57.1	2.4· 10^-13^	0.61
Logistic Regression	Training	87.7	0.0	0.95
Logistic Regression	Test	72.9	1.8· 10^-103^	0.81
Multilayer Perceptron	Training	100.0	0.0	1.0
Multilayer Perceptron	Test	78.2	2.0· 10^-93^	0.53
Naive Bayes	Training	72.8	4.5· 10^-171^	0.69
Naive Bayes	Test	69.3	4.8· 10^-54^	0.62
Random Forest	Training	73.7	1.2· 10^-237^	0.82
Random Forest	Test	57.9	3.3· 10^-15^	0.62
Support Vector Machine	Training	91.3	0.0	0.96
Support Vector Machine	Test	78.5	8.9· 10^-158^	0.87

Results represent averages of 100 training and testing cycles based on random splits of families. The families were split into training and test sets at a ratio of 67:33. Average test significance levels were calculated by averaging logarithms; zero values mean the values are too small to be assessed. The accuracies were weighted by the case-to-control ratio.

The SVM classifier outperforms a *de novo* risk score based on *de novo* variants extracted from the same dataset reported in [17], which achieved p = 5·10^-12^. The accuracy of the SVM classifier is very high bearing in mind the majority of epidemiological studies predict an environmental contribution higher than its error margin [4]. These results validate the decision to remove reference variants from the analysis.

The good performance of the SVM model indicates that the cases and the controls can be separated by a hyperplane in the feature space and hence applying non-linear models such as the MP (neural network) is not necessary. The linearity of the model fits well with the results of the epidemiological analysis [4] indicating the dominance of additive effects over nonlinear ones. 

### GO term overrepresentation analysis

**Fig 2:**
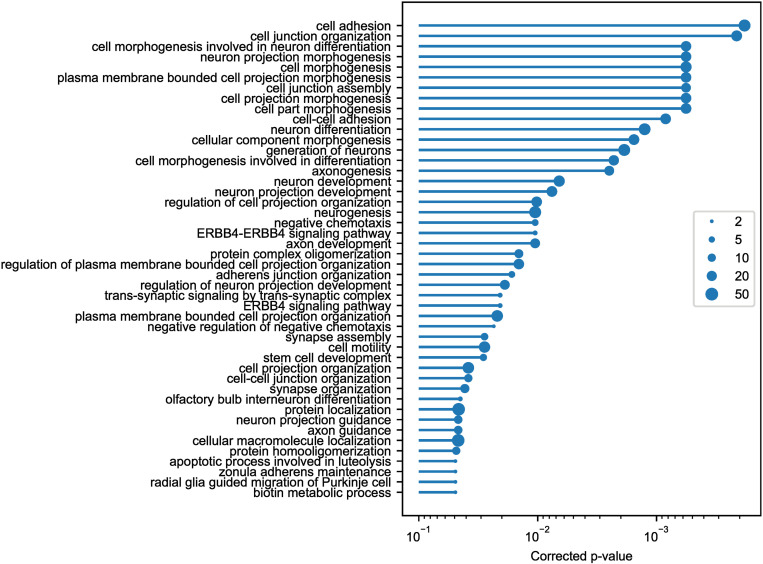
Results of GO-Term analysis for the risk genes identified by the pair search. Overrepresented GO terms were ordered by significance level. The significance was assessed using the hypergeometric test with a minimum significance level of 5% after FDR correction. The circle sizes indicate the number of risk genes associated with each term. Terms associated with more than 50 risk genes were omitted.

The risk genes identified by the pair search were assessed to identify overrepresented GO terms. 46 terms met the adjusted significance level of 0.05 (Fig 2). Terms associated with more than 50 risk genes were omitted. The entire list can be found in the Supporting Information S4 Table. Many of the overrepresented terms are directly related to the development of the CNS: neuron projection morphogenesis, neuron differentiation, axonogenesis, synapse assembly, axon guidance and radial glia guided migration of Purkinje cells. Such results suggest that ASD is a development disorder of the CNS. While it is known that some of the genes associated with ASD play a role in CNS development [5,18–20], the current study can explain over 78% of samples with an AUC of 0.87 and therefore it provides a better basis to generalize the hypothesis.

On the other hand, the presence of “biotin metabolic process” on the list of overrepresented terms is unexpected in the context of ASD etiology. Besides the two associated genes *BTD* and *HLCS*, the risk genes include another gene closely related to biotin, namely *ACACB*, which encodes one of the five enzymes dependent on biotinylation. Both *ACACB* and *HLCS* interact statistically with the *ROBO1* gene responsible for axon guidance. The possible relationship between biotin metabolism and axon guidance requires further study.

The list of the overrepresented terms, the corrected p-values and the associated genes is given in Supporting Information S4 Table.

### Clustering

**Fig 3:**
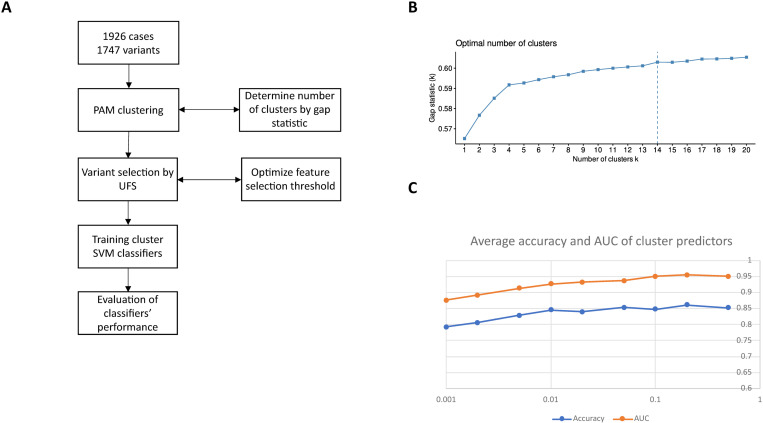
Clustering samples and variant selection. **(A)** Diagram of the data flow. **(B)** Results of the gap statistics for PAM clusters for K = 1..20 indicating that the optimum number of clusters is 14. **(C)** Average weighted accuracy AUC for FPR thresholds 0.001…0.5. Predictors based on PAM clusters achieved the maximum AUC of 0.95 for FPR = 0.2 with an average accuracy of 86.0%.

The first step of the clustering is to determine the number of clusters using the gap statistics (Fig 3A). The gap statistics method was applied to PAM clusters within a range of K from 1 to 20. The optimal number of clusters sought corresponds to the first local maximum of the gap statistics curve, which gives the value 14 (Fig 3B).

The samples were then clustered using PAM based on the determined number of clusters.

In the next step, the set of risk variants identified by the pair search was reduced for each of the 14 clusters using UFS. This step was repeated for FPR thresholds in the range 0.001...0.5. For each FPR value and its corresponding reduced set of variants, per-cluster SVM classifiers were trained and evaluated. The procedure of training and testing the SVM predictors is similar to the one documented earlier for the entire dataset. Only the cases are clustered and other family members are assigned to the same cluster as the corresponding case. Each cluster is randomly split into training and test datasets comprising whole families. The FPR of 0.2 maximizes both the AUC and accuracy of the predictors on the test datasets (Fig 3C). Notably, the cluster predictors exceeded the AUC and the accuracy of the original SVM predictor for the entire dataset with the caveat that no attempt to estimate the clustering error was made. The decline in performance for low FPR values is caused by the removal of significant features, while the poorer performance for high FPR can be attributed to overtraining.

In order to better understand each of the clusters, the associated variants were assigned to genes, except those in intergenic areas and gene overlaps. The resulting genes were processed with the tool BiNGO which looked for overrepresented GO-Terms. The list of genes assigned to each cluster is available in Supporting Information S1 File. Table 4 lists the selected terms associated with each cluster. 5 out of 14 clusters had no overrepresented terms. For the sake of brevity, only the terms related to the development of the nervous system are shown. Overrepresented parents of other overrepresented terms were also omitted. Many of the overrepresented terms given in Table 4 were not identified by an earlier analysis of the entire dataset, for example “presynaptic membrane assembly”, “tangential migration from the subventricular zone to the olfactory bulb” and “axon midline choice point recognition”. Therefore, the clustering analysis adds value by exposing overrepresented terms which are not otherwise visible when analyzing the entire dataset. In terms of answering the question as to whether the ASD is a mix of several genetic diseases, the results are not conclusive. On the one hand, the clustering is successful in a statistical sense as indicated by the gap statistics and by the good accuracy of cluster case status predictors. GO overrepresentation analysis shows unique overrepresented terms in some clusters. On the other hand, the clusters overlap in terms of variants and genes as indicated by Table 4 and Supporting Information S1 File. Mapping the clusters to genetic diseases requires an understanding of the biological interactions between variants and genes. Some of the interactions are discussed in the following sub-section. However, the biological roles of the majority of the underlying genes have yet to be elucidated.

**Table 4:** Results of dataset clustering analysis.

Cluster	No of samples	No of variants	Selected overrepresented GO-Terms
1	588	697	dendrite development, positive regulation of axonogenesis, axon midline choice point recognition
2	488	651	None
3	648	684	Roundabout signaling pathway, axonogenesis, radial glia guided migration of Purkinje cell
4	604	753	axonogenesis, trans-synaptic signaling
5	668	750	telencephalon cell migration, regulation of excitatory synapse assembly, axonogenesis
6	472	629	olfactory bulb interneuron development
7	624	708	None
8	480	690	None
9	556	683	None
10	460	693	negative regulation of axonogenesis, regulation of excitatory synapse assembly, tangential migration from the subventricular zone to the olfactory bulb, regulation of synaptic transmission glutamatergic, axon guidance, trans-synaptic signaling by trans-synaptic complex, biotin metabolic process, regulation of axon extension, regulation of synaptic vesicle transport
11	508	652	olfactory bulb interneuron differentiation, ERBB4-ERBB4 signaling pathway, trans-synaptic signaling by trans-synaptic complex, positive regulation of synapse assembly, axon midline choice point recognition
12	444	662	negative regulation of axonogenesis, dendrite development, telencephalon cell migration, axonogenesis, ERBB2-ERBB4 signaling pathway, ERBB4-ERBB4 signaling pathway, trans-synaptic signaling by trans-synaptic complex, synapse assembly, neural crest cell development, biotin metabolic process, regulation of axon extension
13	596	701	regulation of excitatory synapse assembly, olfactory bulb interneuron differentiation
14	568	677	None

Characteristics of 14 clusters generated by the analysis summarized in Fig 3. The cluster numbers are arbitrary. The number of samples reflects all SFARI family members allocated to each cluster, i.e., probands, siblings and parents. Variants associated with each cluster were identified by the UFS with an FPR threshold of 0.2. The Selected GO term column only contains terms related to the development of the CNS and those which are not parents of other overrepresented terms. The full list of terms is given in Supporting Information S1 File.

### Gene interactions

The methodology applied detects statistical interactions between loci. Mapping the statistical interactions to biological interactions between genes and their products could shed more light on ASD etiology. By inspecting the list of gene pairs given in Supporting Information S1 Table, we identified two pairs which correspond to the known biological interactions *ROBO1*/*SRGAP2* and *GAD1*/*STXBP1*.

*SRGAP2* acts as an intracellular effector for Slit-Robo signaling in neurons [21]. It can bind to the receptor *ROBO1* when it is bound to its extracellular ligand Slit. *SRGAP2* in turn deactivates Rac1 RhoGTPase locally. As a result, actin polymerizes asymmetrically, leading to movement of the axon away from the Slit cue. *SRGAP2* is thought to play a role in the evolution of the human brain through its paralog *SRGAP2C*, which arose at a time when significant expansion of the neocortex began. *SRGAP2* is also hypothesized to play a role in the development of language [22]. Since the proteins coded by the two genes interact directly, it is plausible that minor changes in their structures, which are individually benign, cumulate and thereby impair the signaling pathway leading to the symptoms of ASD.

The genes *GAD1*(*GAD67*) and *STXBP1*(Muc-18–1) both play a key role in the GABA neurotransmitter synthesis and release cycle. The enzyme *GAD1* catalyzes the synthesis of GABA in the presynaptic neurons [23]. Of the two GAD isoforms *GAD67* and *GAD65*, the former provides the most GABA synthesis during the prenatal and early postnatal period, while the latter is the predominant source of GABA in the adult CNS [24]. Besides being a neurotransmitter, GABA drives multiple aspects of the development of the nervous system, including cell proliferation, migration and synapse formation [25].

The gene *STXBP1* regulates synaptic vesicle fusion by interacting with the SNARE complex responsible for vesicle to membrane docking and fusion [26]. *STXBP1* gene mutations have been implicated in many neurological disorders, including epilepsy, ASD and schizophrenia. The genes *GAD1* and the *STXBP1* do not interact directly, although they both contribute to the GABA synthesis and release cycle in neurons. Therefore, one can envisage a cumulative effect of variants that impact both proteins leading to reduced efficiency of the GABA cycle. Such a hypothesis could be potentially verified in vitro by introducing variants detected by the pair search and measuring the release of GABA.

Since we can directly identify only two biological interactions out of 4673 statistical ones, it makes sense to explore alternative approaches. To some extent, such a result can be attributed to the fact that the biological function of many risk genes identified here has yet to be elucidated. A further insight into possible biological interactions can be obtained by investigating triplets of genes where two of them are known to interact biologically and the third one interacts statistically with the other two, as shown in Table 5.

**Table 5:** Triplets of interacting genes.

Gene 1	Gene 2	Gene 3	Biological interaction
*ERBB4*	*NRG1*	*AC087762.1*	*NRG1*/*ERRB4* signaling has been implicated in development of both GABAergic and glutamatergic neurons and synapse formation [27]
*ERBB4*	*SH3GL2*	*CHST1*	*SH3GL2* is involved in clathrin-mediated endocytosis [28]. *ERBB4* receptors are internalized into neurons by clathrin-mediated endocytosis upon binding their ligands *NRG1* [29].
*DLGAP2*	*UBASH3B*	*CTNNA2*	Protein-protein interaction (PPI) of *DLGAP2* and *UBASH3B* was detected by Yeast Two-Hybrid (Y2H) study [30].
*ATXN3*	*DLGAP2*	*IBA57*	PPI of *ATXN3* and *DLGAP2* was detected by Y2H study [31].
*DLG2*	*DLGAP2*	*LINC01173*	*DLG2* binds to *DLGAP* linking postsynaptic glutamate receptors to the PSD [32].
*GALNT1*	*MUC3A*	*AC005772.1*	Initial step of Mucin-type O-glycosylation
*GALNT2*	*MUC3A*	*AASDH*	Initial step of Mucin-type O-glycosylation
*GALNT1*	*MUC2*	*AC005772.1*	Initial step of Mucin-type O-glycosylation
*GALNT2*	*MUC2*	*AC034195.1*	Initial step of Mucin-type O-glycosylation
*ACACB*	*HLCS*	*ROBO1*	Biotin protein ligase (*HCLS*) biotinylates Acetyl CoA carboxylase 2 (*ACACB*) for fatty acid synthesis [33].
*CBFB*	*MAP2*	*AC005772.1*	PPI of *CBFB* and *MAP2* was detected by mRNA display study [34].
*CBFB*	*RUNX2*	*AC005772.1*	*RUNX2* forms complexes with *CBFB* required for the *RUNX2*-depedent transcriptional activation [35]
*EPHA6*	*VAV3*	*DPP6*	*VAV2* and *VAV3* are required for Eph/Ephrin signaling driven axon guidance [36].
*ROBO2*	*SRGAP2*	*DPP6*	*SRGAP2* acts as intracellular effector for Slit-Robo signaling [21].

The Gene 1 interacts biologically with the Gene 2. Both the Gene 1 and the Gene2 interact statistically with the Gene 3, based on the results of the pair search.

 Such triplets can potentially indicate unknown biological interactions between the three genes.

The receptor *ERRB4* and its ligand *NRG1* regulate many aspects of cortex development, including neuron migration, neurite growth and the formation of synapses [27]. The results of the pair search do not indicate any direct statistical interaction between the two genes. However, they both interact statistically with the lncRNA gene *AC087762.1*, whose biological function is not yet known. The gene *AC087762.1* is expressed in the human prefrontal cortex [37]. It is therefore plausible that all three genes interact during the development of the CNS, which contributes to the etiology of ASD.

Table 4 contains another triplet involving the gene *ERBB4* and two other genes, namely *SH3GL2* and *CHST1*. The gene *SH3GL2* codes Endophilin A1, which is expressed primarily in the brain and is involved in clathrin-mediated endocytosis [28]. The *ERBB4* receptors are internalized into neurons by clathrin-mediated endocytosis upon binding their ligands *NRG1* [29]. Nevertheless, the presence of Endophilin A1 is not mandatory since it can be replaced by other proteins and hence proves the direct interaction between *ERBB4* and *SH3GL2* requires further study. The gene *CHST1* codes the enzyme KSGal6ST responsible for sulfation of galactose within keratan sulfate in early postnatal brains [38]. Keratan sulfate proteoglycans direct hippocampus mossy fiber outgrowth in rats [39]. Keratan sulphate have been found to interact with *ERBB4* by microarray protocol [40].

**Fig 4:**
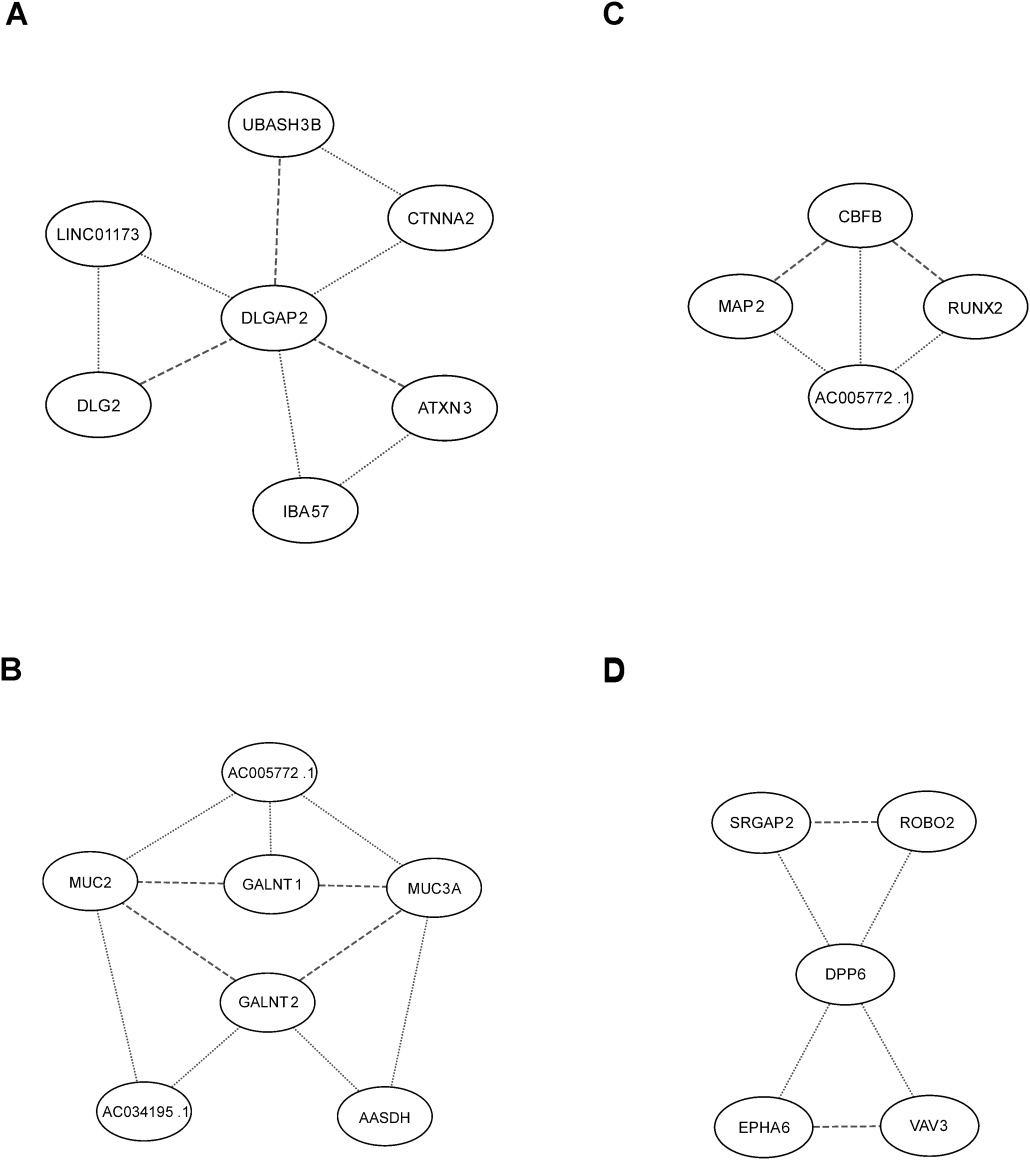
Networks of interacting genes. Dashed lines indicate biological interactions (cf. Table 5). Dotted lines indicate statistical interactions based on the results of the pair search. **(A)** Genes interacting with the PSD genes *DLG2* and *DLGAP2*. **(B)** Interacting genes related to O-glycosylation. **(C)** Interacting genes related to neurite growth. **(D)** Interacting genes related to axonogenesis.

Table 4 contains three entries related to the postsynaptic density (PSD) genes *DLG2* and *DLGAP2*. The resulting network of interacting genes is shown in Fig 4A. The PSD is a highly specialized matrix that is involved in the transmission of neuronal signals across the synaptic junction located in the synaptic terminal of the postsynaptic neurons. *DLGAP2* is involved in synaptic scaling by regulating the turnover of glutamate receptors in response to synaptic activity [32]. *DLG2* binds directly to the *DLGAP2*, linking the postsynaptic glutamate receptors to the PSD. Mutations of the *DLAGP2* gene have been associated with ASD [41]. Two of the interacting genes *ATXN3* and *UBASH3B* can bind directly to the *DLAGP2* on the basis of Y2H studies [30,31]. The gene *ATXN3* codes the deubiquitinating enzyme which regulates protein degradation [42]. *UBASH3B* contains a Ubiquitin-associated (UBA) domain and an SRC homology 3 (SH3) domain. The UBA domain enables the interaction between these proteins and ubiquitin, a protein that plays a critical role in the regulation of protein degradation. The SH3 domain allows *UBASH3B* to bind to proline-rich motifs in other proteins, thereby regulating their activity [43]. Notably, many proteins involved in the PSD, including *DLG2*, also contain the SH3 domain [43]. Therefore, it is likely that both *ATXN3* and *UBASH3B* regulate degradation of *DLGAP2*. The gene *CTNNA2* codes Catenin Alpha 2, which is thought to regulate the morphology of the postsynaptic domains of the excitatory synapses by binding and bundling F-actin [44]. The proposed mechanism of the regulation is recruitment of Catenin Alpha 2 by complexes of N-cadherin responsible for cell-cell adhesion at a synapse with other catenins, leading to dimerization of Catenin Alpha 2. The dimers bind and organize the actin cytoskeleton at the postsynapse. Direct interaction of Catenin Alpha 2 with the PSD proteins is therefore not expected. However, the mutations of *CTNNA2* could impact PSD signaling indirectly through the synapse morphology.

Explaining the role of the other two genes that interact statistically with *DLGAP2*: *IBA57* and *LINC01173* requires further study.

Four of the triplets in Table 4 contain genes related to Mucin-type O-glycosylation (Fig 4B). The network in Fig 4B consists of the two enzymes, *GALNT1* and *GALNT2*, responsible for the initial step of O-glycosylation, together with two of their substrate proteins, *MUC2* and *MUC3A*. The four genes interact statistically in the context of ASD etiology through the intermediate genes *AASDH*, *AC005772.1* and *AC034195.1*. O-glycosylation is a common posttranslational modification of proteins initiated in the Golgi apparatus by a family of ~20 enzymes known as UDP-GalNAc:po-lypeptide N-acetylgalactosaminyltransferases or GALNTs [45]. Mutations of GALNTs have been linked to neurological disorders, including ASD, Alzheimer’s and schizophrenia. In a recent study, *GALNT2* loss of function was linked to a number of developmental and behavioral abnormalities, including autistic traits [46]. However, information about the role of glycosylated proteins in CNS development is still sparse. In one report A CNV of the lncRNA gene *AC005772.1* was linked to ASD [47]. The gene *AC005772.1* is expressed in the cortical plate of human fetal neocortex [48].

The lncRNA gene *AC005772.1* is a part of another network of interacting genes shown in Fig 4C., which includes three other genes, namely *MAP2*, *CBFB* and *RUNX2*. The gene *MAP2* encodes Microtubule-Associated Protein 2 expressed primarily in dendrites and neuron cell bodies [49]. The protein *MAP2* can bind both the microtubules and F-actin. Knockout experiments in mice show that *MAP2* is mainly responsible for dendrite growth and neuron migration, although its presence is not essential. PPI of *MAP2* and *CBFB* were detected by an mRNA display study [34]. *RUNX2* is essential in skeletal development because it regulates osteoblast differentiation and chondrocyte maturation [35]. *RUNX2* forms complexes with the Core-binding Factor-beta protein coded by the gene *CBFB*, which are necessary for *RUNX2*-dependent transcriptional activation [35]. The gene *RUNX2* is also expressed in the hippocampus of the brain, although its function there remains unknown [50]. Recently, *RUNX2* was found to promote neurite growth in pheochromocytoma cells treated with Nerve Growth Factor (NGF) [51]. Explaining the network in Fig 4C requires further study, focusing on the interaction between *MAP2* and *RUNX2* with the lncRNA gene *AC005772.1*.

The gene interaction graph shown in Fig 4D contains four genes that interact statistically with the gene *DPP6*: *EPHA6*, *VAV3*, *SRGAP2* and *ROBO2*. *DPP6* plays two distinct roles in the CNS [52]. Firstly, it associates with A-type K+ channels to control their cellular distribution and gating properties. Secondly, *DPP6* has been found to promote and maintain the growth and stability of filopodia, extending from both dendrites and axons, which help create synaptic connections as well as serve as precursors to dendritic spines. *DPP6* has been linked to a number of neurodevelopmental disorders, including ASD [53]. *EPHA6* belongs to the Eph receptors subfamily of receptor tyrosine kinases. They interact with cell surface-bound ligands Ephrins-A (A1–A6), which are tethered to the plasma membrane via a glycosyl phosphatidyl inositol moiety [54]. Eph/Ephrin signaling is involved in numerous developmental processes, including axon guidance, where it regulates growth cone retraction. As a result, a growing axon avoids an area with a high concentration of Ephrins. An absence of *VAV2* and *VAV3* in mice leads to axon guidance defects that can be attributed to defective endocytosis of the Ephirin-Eph complexes [36]. *SRGAP2* and *ROBO2* belong to the axon guidance pathway and their interaction is similar to that of *ROBO1*/*SRGAP*, described earlier. Therefore, all five interacting genes shown in Fig 4D are involved in axonogenesis, which may explain the detected statistical interactions.

## Conclusion

Analysis of the statistical interactions between loci proves to be an effective method of investigating the etiology of ASD. The new approach addresses a known limitation of the ASD genetics related to the inability to account for common genetic risk given the available dataset sizes. The method identifies 368 novel risk genes. The resulting case status predictors significantly outperform predictors based on *de novo* variants. Gene Ontology analysis identifies multiple overrepresented annotations related to the development of CNS. Clustering the samples increases the predictor accuracy and reveals additional overrepresented terms not visible at the level of the entire dataset. A subset of the detected statistical interactions can be mapped to known biological interactions. Such an analysis can identify potential novel biological interactions and reveal unknown gene functions.

The main limitation of the proposed method is related to the removal of reference variants to reduce the computational complexity. While the contribution of reference variants to ASD risk is likely small, it cannot be completely ruled out.

Future research should focus on the statistical interactions between the risk genes which can be further analyzed experimentally. The methodology applied is likely to work for other complex genetic disorders, such as schizophrenia.

## Supporting information

S1 Table Gene pairs identified by the variant pair search. The table contains names of both genes and the number of variant pairs mapped to a gene pair.
(DOCX)

S2 Table ASD candidate risk genes identified by the pair search. The table contains names, risk scores and functions of the ASD risk genes identified by the pair search. Lack of risk score means a gene is not scored by SFARI.
(XLSX)

S3 Table Results of each training and testing cycle of the SVM classifier. Columns contain model name, phase, number of samples, number of features, true positive count, false negative count, false positive count, true negative count, weighted accuracy, AUC, actual frequency, expected frequency, Chi-Square test probability and its logarithm.
(XLSX)

S4 Table Overrepresented Gene Ontology terms. The table contains a list of overrepresented GO-Terms, the associated p-values and the lists of contributing genes.
(XLSX)

S1 File Overrepresented Gene Ontology terms per each sample cluster. ZIP archive containing over-represented GO-terms for each of the identified clusters. BGO files can be opened with any text editor such as WordPad or Notepad++.
(ZIP)
