# Supplementary material for: Analysis of variant interactions in families with autism points to genes involved in the development of the central nervous system
Source: PLoS One. 2025 Jun 11;20(6):e0326022. doi: 10.1371/journal.pone.0326022 (PMC12157340; doi:10.1371/journal.pone.0326022)
Supplement: S1 Table — The table contains names of both genes and the number of variant pairs mapped to a gene pair. (DOCX) [file pone.0326022.s001.docx]

| Gene | SFARI Score | Main function |
| --- | --- | --- |
| AASDH | - | Covalently binds beta-alanine in an ATP-dependent manner to form a thioester bond with its phosphopantetheine group and transfers it to an, as yet, unknown acceptor. |
| AATF | - | Part of the small subunit (SSU) processome, first precursor of the small eukaryotic ribosomal subunit. |
| ABCA12 | - | Transports lipids such as glucosylceramides from the outer to the inner leaflet of lamellar granules (LGs) membrane, whereby the lipids are finally transported to the keratinocyte periphery via the trans-Golgi network and LGs and released to the apical surface of the granular keratinocytes to form lipid lamellae in the stratum corneum of the epidermis, which is essential for skin barrier function. |
| AC002460.2 | - | Unknown |
| AC004522.6 | - | Unknown |
| AC004805.1 | - | Unknown |
| AC005772.1 | - | Unknown |
| AC008758.4 | - | Unknown |
| AC008780.2 | - | Unknown |
| AC009171.2 | - | Unknown |
| AC009435.1 | - | Unknown |
| AC009975.1 | - | Unknown |
| AC011474.1 | - | Unknown |
| AC012038.1 | - | Unknown |
| AC012533.1 | - | Unknown |
| AC012574.1 | - | Unknown |
| AC013652.1 | - | Unknown |
| AC016074.2 | - | Unknown |
| AC016168.2 | - | Unknown |
| AC016587.1 | - | Unknown |
| AC020687.1 | - | Unknown |
| AC034195.1 | - | Unknown |
| AC041005.1 | - | Unknown |
| AC058822.1 | - | Unknown |
| AC060765.2 | - | Unknown |
| AC068282.1 | - | Unknown |
| AC068631.1 | - | Unknown |
| AC073343.2 | - | Unknown |
| AC079163.2 | - | Unknown |
| AC079380.1 | - | Unknown |
| AC084816.1 | - | Unknown |
| AC087280.2 | - | Unknown |
| AC087762.1 | - | Unknown |
| AC090358.1 | - | Unknown |
| AC090457.1 | - | Unknown |
| AC092598.1 | - | Unknown |
| AC097450.1 | - | Unknown |
| AC100849.1 | - | Unknown |
| AC103718.1 | - | Unknown |
| AC110023.1 | - | Unknown |
| AC113414.1 | - | Unknown |
| AC126755.7 | - | Unknown |
| AC134980.3 | - | Unknown |
| AC139099.2 | - | Unknown |
| AC244230.2 | - | Unknown |
| AC246793.1 | - | Unknown |
| ACACB | - | Mitochondrial enzyme that catalyzes the carboxylation of acetyl-CoA to malonyl-CoA and plays a central role in fatty acid metabolism. Catalyzes a 2-step reaction starting with the ATP-dependent carboxylation of the biotin carried by the biotin carboxyl carrier (BCC) domain followed by the transfer of the carboxyl group from carboxylated biotin to acetyl-CoA. |
| ACP7 | - | Unknown |
| ACSL5 | - | Catalyzes the conversion of long-chain fatty acids to their active form acyl-CoAs for both synthesis of cellular lipids, and degradation via beta-oxidation. |
| ADAM12 | - | Involved in skeletal muscle regeneration, specifically at the onset of cell fusion. Also involved in macrophage-derived giant cells (MGC) and osteoclast formation from mononuclear precursors (By similarity). |
| ADAMTS12 | - | Metalloprotease that may play a role in the degradation of COMP. Cleaves also alpha-2 macroglobulin and aggregan. |
| ADAMTS16 | - | Unknown |
| ADAMTS17 | - | Unknown |
| ADCY3 | 2 | Catalyzes the formation of the signaling molecule cAMP in response to G-protein signaling. Participates in signaling cascades triggered by odorant receptors via its function in cAMP biosynthesis. |
| ADGRD1 | - | Orphan receptor. Signals via G(s)-alpha family of G-proteins. Has a protumorigenic function, especially in glioblastoma. |
| ADGRL2 | - | Calcium-independent receptor of low affinity for alpha- latrotoxin, an excitatory neurotoxin present in black widow spider venom which triggers massive exocytosis from neurons and neuroendocrine cells. |
| ADGRL3 | - | Plays a role in cell-cell adhesion and neuron guidance via its interactions with FLRT2 and FLRT3 that are expressed at the surface of adjacent cells. Plays a role in the development of glutamatergic synapses in the cortex. Important in determining the connectivity rates between the principal neurons in the cortex. |
| ADGRV1 | - | G-protein coupled receptor which has an essential role in the development of hearing and vision. |
| ADSS2 | 2 | Plays an important role in the de novo pathway and in the salvage pathway of purine nucleotide biosynthesis. Catalyzes the first committed step in the biosynthesis of AMP from IMP. |
| AF241726.2 | - | Unknown |
| AF279873.3 | - | Unknown |
| AJ006995.1 | - | Unknown |
| AKAP13 | - | Scaffold protein that plays an important role in assembling signaling complexes downstream of several types of G protein-coupled receptors. |
| AL035415.1 | - | Unknown |
| AL109828.1 | - | Unknown |
| AL133346.1 | - | Unknown |
| AL136456.1 | - | Unknown |
| AL137224.1 | - | Unknown |
| AL139317.5 | - | Unknown |
| AL160272.2 | - | Unknown |
| AL354810.1 | - | Unknown |
| AL356108.1 | - | Unknown |
| AL356295.1 | - | Unknown |
| AL357673.1 | - | Unknown |
| AL359237.1 | - | Unknown |
| AL359436.1 | - | Unknown |
| AL589923.1 | - | Unknown |
| AL596087.2 | - | Unknown |
| AL731769.2 | - | Unknown |
| AL732314.8 | - | Unknown |
| ALKBH3 | - | Dioxygenase that mediates demethylation of DNA and RNA containing 1-methyladenosine (m1A). Repairs alkylated DNA containing 1-methyladenosine (m1A) and 3-methylcytosine (m3C) by oxidative demethylation. |
| ANKH | - | Regulates intra- and extracellular levels of inorganic pyrophosphate (PPi), probably functioning as PPi transporter. |
| ANKRD36C | - | Unknown |
| ANO9 | - | Has calcium-dependent phospholipid scramblase activity; scrambles phosphatidylserine, phosphatidylcholine and galactosylceramide (By similarity). |
| ANXA5 | - | This protein is an anticoagulant protein that acts as an indirect inhibitor of the thromboplastin-specific complex, which is involved in the blood coagulation cascade. |
| AP000446.1 | - | Unknown |
| AP003400.1 | - | Unknown |
| AP005212.4 | - | Unknown |
| APCDD1L | - | Unknown |
| APOA1-AS | - | Unknown |
| ARHGAP25 | - | GTPase activator for the Rho-type GTPases by converting them to an inactive GDP-bound state. |
| ARHGAP6 | - | GTPase activator for the Rho-type GTPases by converting them to an inactive GDP-bound state. Could regulate the interactions of signaling molecules with the actin cytoskeleton. Promotes continuous elongation of cytoplasmic processes during cell motility and simultaneous retraction of the cell body changing the cell morphology. |
| ARID1B | 1,S | Involved in transcriptional activation and repression of select genes by chromatin remodeling (alteration of DNA-nucleosome topology). During neural development a switch from a stem/progenitor to a postmitotic chromatin remodeling mechanism occurs as neurons exit the cell cycle and become committed to their adult state. |
| ARMH4 | - | Unknown |
| ARPP21 | - | Isoform 2 may act as a competitive inhibitor of calmodulin- dependent enzymes such as calcineurin in neurons. |
| ASIC1 | - | Isoform 2 and isoform 3 function as proton-gated sodium channels; they are activated by a drop of the extracellular pH and then become rapidly desensitized. Modulates activity in the circuits underlying innate fear. |
| ASXL1 | - | Probable Polycomb group (PcG) protein involved in transcriptional regulation mediated by ligand-bound nuclear hormone receptors, such as retinoic acid receptors (RARs) and peroxisome proliferator-activated receptor gamma (PPARG). |
| ATF7IP | - | Recruiter that couples transcriptional factors to general transcription apparatus and thereby modulates transcription regulation and chromatin formation. |
| ATXN3 | - | Deubiquitinating enzyme involved in protein homeostasis maintenance, transcription, cytoskeleton regulation, myogenesis and degradation of misfolded chaperone substrates. |
| AZGP1 | 2 | Stimulates lipid degradation in adipocytes and causes the extensive fat losses associated with some advanced cancers. |
| BBS9 | - | The BBSome complex is thought to function as a coat complex required for sorting of specific membrane proteins to the primary cilia. |
| BCKDHB | - | The branched-chain alpha-keto dehydrogenase complex catalyzes the overall conversion of alpha-keto acids to acyl-CoA and CO(2). It contains multiple copies of three enzymatic components: branched-chain alpha-keto acid decarboxylase (E1), lipoamide acyltransferase (E2) and lipoamide dehydrogenase (E3). |
| BEST1 | - | Forms calcium-sensitive chloride channels. Highly permeable to bicarbonate. |
| BMS1P14 | - | Unknown |
| BNC2 | - | Probable transcription factor specific for skin keratinocytes. |
| BTD | - | Catalytic release of biotin from biocytin, the product of biotin-dependent carboxylases degradation. |
| BX890604.2 | - | Unknown |
| C12orf45 | - | Unknown |
| C1orf100 | - | Unknown |
| C1QTNF2 | - | Involved in the regulation of lipid metabolism in adipose tissue and liver. |
| C8orf34 | - | Unknown |
| CA5B | - | Reversible hydration of carbon dioxide. |
| CACNA1D | 2 | Voltage-sensitive calcium channels (VSCC) mediate the entry of calcium ions into excitable cells and are also involved in a variety of calcium-dependent processes, including muscle contraction, hormone or neurotransmitter release, gene expression, cell motility, cell division and cell death. |
| CACNB2 | 2 | Beta subunit of voltage-dependent calcium channels which contributes to the function of the calcium channel by increasing peak calcium current (By similarity). |
| CAMK1D | - | Calcium/calmodulin-dependent protein kinase that operates in the calcium-triggered CaMKK-CaMK1 signaling cascade and, upon calcium influx, activates CREB-dependent gene transcription, regulates calcium- mediated granulocyte function and respiratory burst and promotes basal dendritic growth of hippocampal neurons. |
| CAMSAP3 | - | Key microtubule-organizing protein that specifically binds the minus-end of non-centrosomal microtubules and regulates their dynamics and organization. |
| CAMTA1 | - | Transcriptional activator. |
| CAND2 | - | Probable assembly factor of SCF (SKP1-CUL1-F-box protein) E3 ubiquitin ligase complexes that promotes the exchange of the substrate- recognition F-box subunit in SCF complexes, thereby playing a key role in the cellular repertoire of SCF complexes. |
| CASC11 | - | Unknown |
| CATSPERE | - | Auxiliary component of the CatSper complex, a complex involved in sperm cell hyperactivation. |
| CBFB | - | Forms the heterodimeric complex core-binding factor (CBF) with RUNX family proteins (RUNX1, RUNX2, and RUNX3). |
| CBR4 | - | Component of the heterotetramer complex KAR (3-ketoacyl-[acyl carrier protein] reductase or 3-ketoacyl-[ACP] reductase) that forms part of the mitochondrial fatty acid synthase (mtFAS). |
| CBS | - | Hydro-lyase catalyzing the first step of the transsulfuration pathway, where the hydroxyl group of L-serine is displaced by L- homocysteine in a beta-replacement reaction to form L-cystathionine, the precursor of L-cysteine. This catabolic route allows the elimination of L-methionine and the toxic metabolite L-homocysteine. Also involved in the production of hydrogen sulfide, a gasotransmitter with signaling and cytoprotective effects on neurons. |
| CCDC186 | - | Unknown |
| CCDC68 | - | Centriolar protein required for centriole subdistal appendage assembly and microtubule anchoring in interphase cells. |
| CCDC7 | - | May play a role in tumorigenesis. |
| CCND3 | - | Regulatory component of the cyclin D3-CDK4 (DC) complex that phosphorylates and inhibits members of the retinoblastoma (RB) protein family including RB1 and regulates the cell-cycle during G(1)/S transition. |
| CCNJL | - | Unknown |
| CCNT2-AS1 | - | Unknown |
| CCSER1 | 2 | Unknown |
| CCT3 | - | Component of the chaperonin-containing T-complex (TRiC), a molecular chaperone complex that assists the folding of proteins upon ATP hydrolysis. |
| CD226 | - | Involved in intercellular adhesion, lymphocyte signaling, cytotoxicity and lymphokine secretion mediated by cytotoxic T- lymphocyte (CTL) and NK cell. Cell surface receptor for NECTIN2. |
| CD81-AS1 | - | Unknown |
| CD96 | - | May be involved in adhesive interactions of activated T and NK cells during the late phase of the immune response. |
| CD99 | - | Involved in T-cell adhesion processes and in spontaneous rosette formation with erythrocytes. |
| CDC34 | - | Accepts ubiquitin from the E1 complex and catalyzes its covalent attachment to other proteins. |
| CDC42EP4 | - | Probably involved in the organization of the actin cytoskeleton. May act downstream of CDC42 to induce actin filament assembly leading to cell shape changes. Induces pseudopodia formation, when overexpressed in fibroblasts. |
| CDH13 | 2 | Cadherins are calcium-dependent cell adhesion proteins. They preferentially interact with themselves in a homophilic manner in connecting cells; cadherins may thus contribute to the sorting of heterogeneous cell types. May act as a negative regulator of neural cell growth. |
| CDH4 | - | Cadherins are calcium-dependent cell adhesion proteins. They preferentially interact with themselves in a homophilic manner in connecting cells; cadherins may thus contribute to the sorting of heterogeneous cell types. May play an important role in retinal development. |
| CDH8 | 2 | Cadherins are calcium-dependent cell adhesion proteins. They preferentially interact with themselves in a homophilic manner in connecting cells; cadherins may thus contribute to the sorting of heterogeneous cell types. |
| CFAP44 | - | Flagellar protein involved in sperm flagellum axoneme organization and function. |
| CHST1 | - | Sulfotransferase that utilizes 3'-phospho-5'-adenylyl sulfate (PAPS) as sulfonate donor to catalyze the transfer of sulfate to position 6 of internal galactose (Gal) residues of keratan. Involved in biosynthesis of phosphacan, a major keratan sulfate proteoglycan in the developing brain. |
| CLIP2 | 3 | Seems to link microtubules to dendritic lamellar body (DLB), a membranous organelle predominantly present in bulbous dendritic appendages of neurons linked by dendrodendritic gap junctions. |
| CNN3 | - | Thin filament-associated protein that is implicated in the regulation and modulation of smooth muscle contraction. |
| CNOT4 | - | Has E3 ubiquitin ligase activity, promoting ubiquitination and degradation of target proteins. |
| CNTLN | - | Required for centrosome cohesion and recruitment of CEP68 to centrosomes. |
| CNTN6 | 2 | Contactins mediate cell surface interactions during nervous system development. |
| CNTNAP3B | - | Unknown |
| CNTROB | - | Required for centriole duplication. |
| CPNE5 | - | Probable calcium-dependent phospholipid-binding protein that may play a role in calcium-mediated intracellular processes. Plays a role in dendrite formation by melanocytes. |
| CR381653.1 | - | Unknown |
| CRACD | - | Involved in epithelial cell integrity by acting on the maintenance of the actin cytoskeleton. |
| CRISPLD2 | - | Promotes matrix assembly. |
| CTNNA2 | S | May function as a linker between cadherin adhesion receptors and the cytoskeleton to regulate cell-cell adhesion and differentiation in the nervous system. Required for proper regulation of cortical neuronal migration and neurite growth. |
| CTU1 | - | Plays a central role in 2-thiolation of mcm(5)S(2)U at tRNA wobble positions of tRNA(Lys), tRNA(Glu) and tRNA(Gln). |
| CYB561 | - | Transmembrane reductase that uses ascorbate as an electron donor in the cytoplasm and transfers electrons across membranes to reduce monodehydro-L-ascorbate radical in the lumen of secretory vesicles. |
| CYP20A1 | - | Unknown |
| CYP46A1 | - | P450 monooxygenase that plays a major role in cholesterol homeostasis in the brain. |
| DAB1 | - | Adapter molecule functioning in neural development. |
| DANT2 | - | Unknown |
| DCLK1 | - | Probable kinase that may be involved in a calcium-signaling pathway controlling neuronal migration in the developing brain. |
| DEFB125 | - | Has antibacterial activity. |
| DEK | - | Involved in chromatin organization. |
| DELE1 | - | Key activator of the integrated stress response (ISR) following mitochondrial stress |
| DENND4C | - | Guanine nucleotide exchange factor (GEF) activating RAB10. Promotes the exchange of GDP to GTP, converting inactive GDP-bound RAB10 into its active GTP-bound form. |
| DGKI | 3 | Diacylglycerol kinase that converts diacylglycerol/DAG into phosphatidic acid/phosphatidate/PA and regulates the respective levels of these two bioactive lipids. May play a role in presynaptic diacylglycerol/DAG signaling and control neurotransmitter release during metabotropic glutamate receptor-dependent long-term depression. |
| DLG2 | 2 | Involved in regulation of synaptic stability at cholinergic synapses. |
| DLGAP2 | 2 | May play a role in the molecular organization of synapses and neuronal cell signaling. |
| DNAAF2 | - | Required for cytoplasmic pre-assembly of axonemal dyneins, thereby playing a central role in motility in cilia and flagella. |
| DNAH11 | - | Produces force towards the minus ends of microtubules. |
| DNAJC27-AS1 | - | Unknown |
| DPP6 | 2 | Promotes cell surface expression of the potassium channel KCND2. |
| EAF2 | - | Acts as a transcriptional transactivator of TCEA1 elongation activity (By similarity). |
| EBF2 | - | Transcription factor that, in osteoblasts, activates the decoy receptor for RANKL, TNFRSF11B, which in turn regulates osteoclast differentiation. |
| ECHDC2 | - | Unknown |
| EDA | - | Cytokine which is involved in epithelial-mesenchymal signaling during morphogenesis of ectodermal organs |
| EDNRA | - | Receptor for endothelin-1. |
| EHBP1 | - | May play a role in actin reorganization. |
| EML6 | - | May modify the assembly dynamics of microtubules, such that microtubules are slightly longer, but more dynamic. |
| ENPEP | - | Regulates central hypertension through its calcium-modulated preference to cleave N-terminal acidic residues from peptides such as angiotensin II. |
| EPB41 | - | Recruits DLG1 to membranes. |
| EPHA6 | - | Receptor tyrosine kinase which binds promiscuously GPI- anchored ephrin-A family ligands residing on adjacent cells, leading to contact-dependent bidirectional signaling into neighboring cells. Predicted to be involved in axon guidance. |
| EPS8L1 | - | Stimulates guanine exchange activity of SOS1. May play a role in membrane ruffling and remodeling of the actin cytoskeleton. |
| ERBB4 | - | Acts as cell-surface receptor for the neuregulins NRG1, NRG2, NRG3 and NRG4 and the EGF family members BTC, EREG and HBEGF. |
| ERC1 | - | Regulatory subunit of the IKK complex. May be involved in the organization of the cytomatrix at the nerve terminals active zone (CAZ) which regulates neurotransmitter release. |
| ESRRG | - | Orphan receptor that acts as transcription activator in the absence of bound ligand. |
| ETV6 | - | Transcriptional repressor; binds to the DNA sequence 5'- CCGGAAGT-3'. |
| FAF1 | - | Ubiquitin-binding protein. |
| FAM117B | - | Unknown |
| FAM13C | - | Unknown |
| FAM149A | - | Unknown |
| FAM172A | - | Plays a role in the regulation of alternative splicing, by interacting with AGO2 and CHD7. |
| FAM189A2 | - | Unknown |
| FAM242F | - | Unknown |
| FAM66D | - | Unknown |
| FAM83E | - | May play a role in MAPK signaling. |
| FARP1 | - | Functions as guanine nucleotide exchange factor for RAC1. Plays a role in the assembly and disassembly of dendritic filopodia, the formation of dendritic spines, regulation of dendrite length and ultimately the formation of synapses. |
| FASTKD1 | - | Involved in the down-regulation of mitochondrial MT-ND3 mRNA levels which leads to decreased respiratory complex I abundance and activity. |
| FBXO33 | 2 | Substrate recognition component of a SCF (SKP1-CUL1-F-box protein) E3 ubiquitin-protein ligase complex which mediates the ubiquitination and subsequent proteasomal degradation of target proteins. |
| FER1L6 | - | Unknown |
| FGF2 | - | Acts as a ligand for FGFR1, FGFR2, FGFR3 and FGFR4. Also acts as an integrin ligand which is required for FGF2 signaling. |
| FMN2 | - | Actin-binding protein that is involved in actin cytoskeleton assembly and reorganization. |
| FO393418.1 | - | Unknown |
| FP236383.3 | - | Unknown |
| FRAS1 | - | Involved in extracellular matrix organization. Involved in brain organization and function. |
| FRG1-DT | - | Unknown |
| FRG2C | - | Unknown |
| FRMD4A | - | Scaffolding protein that regulates epithelial cell polarity by connecting ARF6 activation with the PAR3 complex. |
| FRMPD2 | - | May play a role in the regulation of tight junction formation. Binds phosphatidylinositol 3,4-bisphosphate (PtdIns(3,4)P2). |
| FST | - | Binds directly to activin and functions as an activin antagonist. |
| GAD1 | - | Catalyzes the synthesis of the inhibitory neurotransmitter gamma-aminobutyric acid (GABA) with pyridoxal 5'-phosphate as cofactor. |
| GALNT1 | - | Catalyzes the initial reaction in O-linked oligosaccharide biosynthesis, the transfer of an N-acetyl-D-galactosamine residue to a serine or threonine residue on the protein receptor. |
| GALNT2 | S | Catalyzes the initial reaction in O-linked oligosaccharide biosynthesis, the transfer of an N-acetyl-D-galactosamine residue to a serine or threonine residue on the protein receptor. |
| GPI | - | In the cytoplasm, catalyzes the conversion of glucose-6- phosphate to fructose-6-phosphate, the second step in glycolysis, and the reverse reaction during gluconeogenesis. |
| GRAMD1B | - | Cholesterol transporter that mediates non-vesicular transport of cholesterol from the plasma membrane to the endoplasmic reticulum. |
| GREB1 | - | May play a role in estrogen-stimulated cell proliferation. |
| GRIP1 | 2 | May play a role as a localized scaffold for the assembly of a multiprotein signaling complex and as mediator of the trafficking of its binding partners at specific subcellular location in neurons. Through complex formation with NSG1, GRIA2 and STX12 controls the intracellular fate of AMPAR. |
| HDAC9 | - | Responsible for the deacetylation of lysine residues on the N-terminal part of the core histones (H2A, H2B, H3 and H4). Protects neurons from apoptosis, both by inhibiting JUN phosphorylation by MAPK10 and by repressing JUN transcription via HDAC1 recruitment to JUN promoter. |
| HDLBP | 1 | Appears to play a role in cell sterol metabolism. |
| HEATR5A | - | Unknown |
| HEATR5B | - | Component of clathrin-coated vesicles. |
| HECW1 | - | E3 ubiquitin-protein ligase that mediates ubiquitination and subsequent degradation of DVL1. |
| HIVEP1 | - | This protein specifically binds to the DNA sequence 5'- GGGACTTTCC-3' which is found in the enhancer elements of numerous viral promoters such as those of SV40, CMV, or HIV-1. |
| HLA-DQA1 | - | Binds peptides derived from antigens that access the endocytic route of antigen presenting cells (APC) and presents them on the cell surface for recognition by the CD4 T-cells. |
| HLA-DRB1 | 2 | A beta chain of antigen-presenting major histocompatibility complex class II (MHCII) molecule. |
| HLCS | - | Biotin--protein ligase catalyzing the biotinylation of the 4 biotin-dependent carboxylases acetyl-CoA-carboxylase, pyruvate carboxylase, propionyl-CoA carboxylase, and methylcrotonyl-CoA carboxylase. |
| IBA57 | - | Involved in the maturation of mitochondrial 4Fe-4S proteins functioning late in the iron-sulfur cluster assembly pathway. |
| IL1RAPL1 | 2 | May regulate secretion and presynaptic differentiation through inhibition of the activity of N-type voltage-gated calcium channel. |
| IL21R | - | This is a receptor for interleukin-21. |
| IQCB1 | - | Involved in ciliogenesis. |
| ITPK1 | - | Kinase that can phosphorylate various inositol polyphosphate such as Ins(3,4,5,6)P4 or Ins(1,3,4)P3. |
| KANSL1L | - | Unknown |
| KATNB1 | - | Participates in a complex which severs microtubules in an ATP-dependent manner. Microtubule release within the cell body of neurons may be required for their transport into neuronal processes by microtubule-dependent motor proteins. This transport is required for axonal growth. |
| KCNB1 | 1,S | Voltage-gated potassium channel that mediates transmembrane potassium transport in excitable membranes, primarily in the brain, but also in the pancreas and cardiovascular system. |
| KCNB2 | - | Voltage-gated potassium channel that mediates transmembrane potassium transport in excitable membranes, primarily in the brain and smooth muscle cells. |
| KCNG2 | - | Potassium channel subunit. |
| KCNS3 | 2 | Potassium channel subunit that does not form functional channels by itself. |
| KDM1B | 2 | Histone demethylase that demethylates 'Lys-4' of histone H3, a specific tag for epigenetic transcriptional activation, thereby acting as a corepressor. |
| KIAA0040 | - | Unknown |
| KIAA1324 | - | Unknown |
| KIFC3 | - | Minus-end microtubule-dependent motor protein. |
| KLF12 | - | Confers strong transcriptional repression to the AP-2-alpha gene. |
| KMT2E | 1,S | Associates with chromatin regions downstream of transcriptional start sites of active genes and thus regulates gene transcription. Chromatin interaction is mediated via the binding to tri-methylated histone H3 at 'Lys-4' (H3K4me3). |
| L1CAM | - | Neural cell adhesion molecule involved in the dynamics of cell adhesion and in the generation of transmembrane signals at tyrosine kinase receptors. During brain development, critical in multiple processes, including neuronal migration, axonal growth and fasciculation, and synaptogenesis. In the mature brain, plays a role in the dynamics of neuronal structure and function, including synaptic plasticity. |
| LIMS1 | - | Adapter protein in a cytoplasmic complex linking beta- integrins to the actin cytoskeleton, bridges the complex to cell surface receptor tyrosine kinases and growth factor receptors. |
| LIN54 | - | Component of the DREAM complex, a multiprotein complex that can both act as a transcription activator or repressor depending on the context. |
| LINC00163 | - | Unknown |
| LINC00229 | - | Unknown |
| LINC00539 | - | Unknown |
| LINC00618 | - | Unknown |
| LINC00922 | - | Unknown |
| LINC01107 | - | Unknown |
| LINC01169 | - | Unknown |
| LINC01173 | - | Unknown |
| LINC01197 | - | Unknown |
| LINC01234 | - | Unknown |
| LINC01237 | - | Unknown |
| LINC01290 | - | Unknown |
| LINC01378 | - | Unknown |
| LINC01476 | - | Unknown |
| LINC01483 | - | Unknown |
| LINC01515 | - | Unknown |
| LINC01541 | - | Unknown |
| LINC01811 | - | Unknown |
| LINC01814 | - | Unknown |
| LINC02008 | - | Unknown |
| LINC02062 | - | Unknown |
| LINC02109 | - | Unknown |
| LINC02141 | - | Unknown |
| LINC02208 | - | Unknown |
| LINC02210-CRHR1 | - | Unknown |
| LINC02241 | - | Unknown |
| LINC02267 | - | Unknown |
| LINC02291 | - | Unknown |
| LINC02317 | - | Unknown |
| LINC02336 | - | Unknown |
| LINC02343 | - | Unknown |
| LINC02542 | - | Unknown |
| LMF1 | - | Involved in the maturation of specific proteins in the endoplasmic reticulum. |
| LPP | - | May play a structural role at sites of cell adhesion in maintaining cell shape and motility. |
| LRRK2 | - | Serine/threonine-protein kinase which phosphorylates a broad range of proteins involved in multiple processes such as neuronal plasticity, innate immunity, autophagy, and vesicle trafficking |
| LRRTM4 | - | May play a role in the development and maintenance of the vertebrate nervous system. Exhibits strong synaptogenic activity, restricted to excitatory presynaptic differentiation. |
| MACROD2 | 2 | Removes ADP-ribose from aspartate and glutamate residues in proteins bearing a single ADP-ribose moiety. |
| MAEA | - | Core component of the CTLH E3 ubiquitin-protein ligase complex that selectively accepts ubiquitin from UBE2H and mediates ubiquitination and subsequent proteasomal degradation of the transcription factor HBP1. |
| MAJIN | - | Meiosis-specific telomere-associated protein involved in meiotic telomere attachment to the nucleus inner membrane, a crucial step for homologous pairing and synapsis. |
| MAP2 | - | The proteins of this family are thought to be involved in microtubule assembly, which is an essential step in neurogenesis. The products of similar genes in rat and mouse are neuron-specific cytoskeletal proteins that are enriched in dendrites. |
| MAP3K20 | - | Stress-activated component of a protein kinase signal transduction cascade that promotes programmed cell death in response to various stress, such as ribosomal stress, osmotic shock and ionizing radiation. |
| MAST3 | 3 | Unknown |
| MIR4300HG | - | Unknown |
| MPP4 | - | May play a role in retinal photoreceptors development. |
| MTERF1 | - | Transcription termination factor. |
| MTFMT | - | Methionyl-tRNA formyltransferase that formylates methionyl- tRNA in mitochondria and is crucial for translation initiation. |
| MUC2 | - | Coats the epithelia of the intestines and other mucus membrane-containing organs to provide a protective, lubricating barrier against particles and infectious agents at mucosal surfaces. |
| MUC3A | - | Major glycoprotein component of a variety of mucus gels. |
| MVB12A | - | Component of the ESCRT-I complex, a regulator of vesicular trafficking process. |
| MYH11 | - | Muscle contraction. |
| MYOF | - | Calcium/phospholipid-binding protein that plays a role in the plasmalemma repair mechanism of endothelial cells that permits rapid resealing of membranes disrupted by mechanical stress. |
| NAALADL2 | 2 | May be catalytically inactive. |
| NBPF26 | - | Unknown |
| NCOA1 | 1 | Nuclear receptor coactivator that directly binds nuclear receptors and stimulates the transcriptional activities in a hormone- dependent fashion. |
| NFATC1 | - | Plays a role in the inducible expression of cytokine genes in T-cells, especially in the induction of the IL-2 or IL-4 gene transcription. Also controls gene expression in embryonic cardiac cells. |
| NIM1K | - | Unknown |
| NME7 | - | Major role in the synthesis of nucleoside triphosphates other than ATP. |
| NR6A1 | - | Orphan nuclear receptor. |
| NRG1 | - | Direct ligand for ERBB3 and ERBB4 tyrosine kinase receptors. |
| NRG3 | - | Direct ligand for the ERBB4 tyrosine kinase receptor. |
| NRXN1 | 1 | Neuronal cell surface protein involved in cell recognition and cell adhesion by forming intracellular junctions through binding to neuroligins. Plays a role in formation of synaptic junctions. |
| NSF | - | Required for vesicle-mediated transport. Catalyzes the fusion of transport vesicles within the Golgi cisternae. |
| NSMAF | - | Couples the p55 TNF-receptor (TNF-R55 / TNFR1) to neutral sphingomyelinase (N-SMASE). |
| NT5DC3 | - | Unknown |
| NUP205 | - | Plays a role in the nuclear pore complex (NPC) assembly and/or maintenance. |
| NXPH1 | 2 | May be signaling molecules that resemble neuropeptides and that act by binding to alpha-neurexins and possibly other receptors. |
| OR3A2 | - | Odorant receptor. |
| OR9G1 | - | Odorant receptor. |
| OSBP2 | - | Binds 7-ketocholesterol. |
| OTUD7A | 2 | Has deubiquitinating activity towards 'Lys-11'-linked polyubiquitin chains. |
| PARD3 | - | Adapter protein involved in asymmetrical cell division and cell polarization processes. Required for establishment of neuronal polarity and normal axon formation in cultured hippocampal neurons. |
| PARP16 | - | Intracellular mono-ADP-ribosyltransferase that plays a role in different processes, such as protein translation and unfolded protein response (UPR), through the mono-ADP-ribosylation of proteins involved in those processes. |
| PCCA | S | This is one of the 2 subunits of the biotin-dependent propionyl-CoA carboxylase (PCC), a mitochondrial enzyme involved in the catabolism of odd chain fatty acids, branched-chain amino acids isoleucine, threonine, methionine, and valine and other metabolites. |
| PCMTD1 | - | Substrate recognition component of an ECS (Elongin BC-CUL5- SOCS-box protein) E3 ubiquitin ligase complex which mediates the ubiquitination and subsequent proteasomal degradation of target proteins. |
| PGM5P4-AS1 | - | Unknown |
| PHYH | - | Catalyzes the 2-hydroxylation of not only racemic phytanoyl- CoA and the isomers of 3-methylhexadecanoyl-CoA, but also a variety of other mono-branched 3-methylacyl-CoA esters (with a chain length of at least seven carbon atoms) and straight-chain acyl-CoA esters (with a chain length longer than four carbon atoms). |
| PINX1 | - | Microtubule-binding protein essential for faithful chromosome segregation. |
| PLCD4 | 2 | Hydrolyzes the phosphatidylinositol 4,5-bisphosphate (PIP2) to generate 2 second messenger molecules diacylglycerol (DAG) and inositol 1,4,5-trisphosphate (IP3) |
| PLPP7 | - | Plays a role as negative regulator of myoblast differentiation, in part through effects on MTOR signaling. |
| PMM2 | - | Involved in the synthesis of the GDP-mannose and dolichol- phosphate-mannose required for a number of critical mannosyl transfer reactions. |
| PNPLA1 | - | Omega-hydroxyceramide transacylase involved in the synthesis of omega-O-acylceramides (esterified omega-hydroxyacyl-sphingosine; EOS), which are extremely hydrophobic lipids involved in skin barrier formation. |
| PNPT1 | - | RNA-binding protein implicated in numerous RNA metabolic processes. |
| POTEI | - | Involved in retina homeostasis and in axonogensis. |
| PPAT | - | Catalyzes the formation of phosphoribosylamine from phosphoribosylpyrophosphate (PRPP) and glutamine. |
| PPP1R12C | - | Regulates myosin phosphatase activity. |
| PPP2R5C | - | The B regulatory subunit might modulate substrate selectivity and catalytic activity, and also might direct the localization of the catalytic enzyme to a particular subcellular compartment. |
| PRKCB | 2 | Calcium-activated, phospholipid- and diacylglycerol (DAG)- dependent serine/threonine-protein kinase involved in various cellular processes such as regulation of the B-cell receptor (BCR) signalosome, oxidative stress-induced apoptosis, androgen receptor-dependent transcription regulation, insulin signaling and endothelial cells proliferation. |
| PTPRD | - | Can bidirectionally induce pre- and post-synaptic differentiation of neurons by mediating interaction with IL1RAP and IL1RAPL1 trans-synaptically. Involved in pre-synaptic differentiation through interaction with SLITRK2. |
| QDPR | - | Catalyzes the conversion of quinonoid dihydrobiopterin into tetrahydrobiopterin. |
| RAB26 | - | The small GTPases Rab are key regulators of intracellular membrane trafficking, from the formation of transport vesicles to their fusion with membranes. |
| RASA4B | - | Ca(2+)-dependent Ras GTPase-activating protein, that may play a role in the Ras-MAPK pathway. |
| RETREG1 | - | Endoplasmic reticulum (ER)-anchored autophagy regulator which mediates ER delivery into lysosomes through sequestration into autophagosomes. |
| RNF103-CHMP3 | - | Unknown |
| RNF17 | - | Seems to be involved in regulation of transcriptional activity of MYC. In vitro, inhibits DNA-binding activity of Mad-MAX heterodimers. |
| RNF4 | - | E3 ubiquitin-protein ligase which binds polysumoylated chains covalently attached to proteins and mediates 'Lys-6'-, 'Lys-11'-, 'Lys- 48'- and 'Lys-63'-linked polyubiquitination of those substrates and their subsequent targeting to the proteasome for degradation. |
| ROBO1 | - | Receptor for SLIT1 and SLIT2 that mediates cellular responses to molecular guidance cues in cellular migration, including axonal navigation at the ventral midline of the neural tube and projection of axons to different regions during neuronal development. |
| ROBO2 | 2 | Receptor for SLIT2, and probably SLIT1, which are thought to act as molecular guidance cue in cellular migration, including axonal navigation at the ventral midline of the neural tube and projection of axons to different regions during neuronal development. |
| RPS5 | - | Component of the small ribosomal subunit. |
| RPS9 | - | Component of the small ribosomal subunit. |
| RUNX2 | - | Transcription factor involved in osteoblastic differentiation and skeletal morphogenesis. |
| RXFP1 | - | Receptor for relaxins. |
| SCN11A | - | This protein mediates the voltage-dependent sodium ion permeability of excitable membranes. |
| SEC31A | - | Component of the coat protein complex II (COPII) which promotes the formation of transport vesicles from the endoplasmic reticulum (ER). |
| SEMA3E | - | Plays an important role in signaling via the cell surface receptor PLXND1. Plays an important role in ensuring the specificity of synapse formation. |
| SEMA4A | - | Cell surface receptor for PLXNB1, PLXNB2, PLXNB3 and PLXND1 that plays an important role in cell-cell signaling. Regulates glutamatergic and GABAergic synapse development. Promotes axon growth cone collapse. Inhibits axonal extension by providing local signals to specify territories inaccessible for growing axons. |
| SGCB | - | Component of the sarcoglycan complex, a subcomplex of the dystrophin-glycoprotein complex which forms a link between the F-actin cytoskeleton and the extracellular matrix. |
| SH3GL2 | - | Implicated in synaptic vesicle endocytosis. |
| SH3RF2 | - | Has E3 ubiquitin-protein ligase activity. |
| SLC10A7 | - | Has an essential role in the biosynthesis and trafficking of glycosaminoglycans and glycoproteins, to produce a proper functioning extracellular matrix. |
| SLC19A1 | - | Antiporter that mediates the import of reduced folates or a subset of cyclic dinucleotides, driven by the export of organic anions. |
| SLC22A16 | - | High affinity carnitine transporter; the uptake is partially sodium-ion dependent. |
| SLC39A11 | - | Functions as a cellular zinc transporter. |
| SLIT3 | - | May act as molecular guidance cue in cellular migration, and function may be mediated by interaction with roundabout homolog receptors. |
| SND1 | 2 | Endonuclease that mediates miRNA decay of both protein-free and AGO2-loaded miRNAs. |
| SNX30 | - | Involved in the regulation of endocytosis and in several stages of intracellular trafficking. |
| SOD2 | - | Destroys superoxide anion radicals which are normally produced within the cells and which are toxic to biological systems. |
| SORBS1 | - | Plays a role in tyrosine phosphorylation of CBL by linking CBL to the insulin receptor. |
| SORCS3 | 2 | Unknown |
| SPATA5 | - | Unknown |
| SPOCK1 | - | May play a role in cell-cell and cell-matrix interactions. May contribute to various neuronal mechanisms in the central nervous system. |
| SPP2 | 2 | Could coordinate an aspect of bone turnover. |
| SRGAP2 | - | Postsynaptic RAC1 GTPase activating protein (GAP) that plays a key role in neuronal morphogenesis and migration mainly during development of the cerebral cortex. |
| SRGAP2B | - | May regulate cell migration and differentiation through interaction with and inhibition of SRGAP2. In contrast to SRGAP2C, it is not able to induce long-lasting changes in synaptic density throughout adulthood. |
| SRRM2-AS1 | - | Unknown |
| SSC5D | - | Binds to extracellular matrix proteins. |
| STK24 | - | Serine/threonine-protein kinase that acts on both serine and threonine residues and promotes apoptosis in response to stress stimuli and caspase activation. |
| STK3 | - | Stress-activated, pro-apoptotic kinase which, following caspase-cleavage, enters the nucleus and induces chromatin condensation followed by internucleosomal DNA fragmentation. |
| STRBP | - | Plays a role in regulation of cell growth. |
| STX17-AS1 | - | Unknown |
| STXBP1 | 1,S | Participates in the regulation of synaptic vesicle docking and fusion through interaction with GTP-binding proteins. |
| SUGCT | - | Catalyzes the succinyl-CoA-dependent conversion of glutarate to glutaryl-CoA. |
| TBCD | - | Tubulin-folding protein implicated in the first step of the tubulin folding pathway and required for tubulin complex assembly. Involved in neuron morphogenesis. |
| TCIM | - | Seems to be involved in the regulation of cell growth and differentiation, may play different and opposite roles depending on the tissue or cell type. May enhance the WNT-CTNNB1 pathway by relieving antagonistic activity of CBY1. Enhances the proliferation of follicular dendritic cells. |
| TDG | - | DNA glycosylase that plays a key role in active DNA demethylation: specifically recognizes and binds 5-formylcytosine (5fC) and 5-carboxylcytosine (5caC) in the context of CpG sites and mediates their excision through base-excision repair (BER) to install an unmethylated cytosine. |
| TDRD3 | - | Scaffolding protein that specifically recognizes and binds dimethylarginine-containing proteins. |
| TFF2 | - | Inhibits gastrointestinal motility and gastric acid secretion. |
| TLN2 | - | As a major component of focal adhesion plaques that links integrin to the actin cytoskeleton, may play an important role in cell adhesion. |
| TMC5 | - | Probable ion channel. |
| TMED3 | - | Potential role in vesicular protein trafficking, mainly in the early secretory pathway. |
| TMEM132B | - | Unknown |
| TMEM51 | - | Unknown |
| TMPRSS2 | - | Plasma membrane-anchored serine protease that cleaves at arginine residues. |
| TNK2 | - | Non-receptor tyrosine-protein and serine/threonine-protein kinase that is implicated in cell spreading and migration, cell survival, cell growth and proliferation. May be involved both in adult synaptic function and plasticity and in brain development. |
| TNS3 | - | Involved in the dissociation of the integrin-tensin-actin complex. |
| TPD52 | - | Unknown |
| TRIM5 | - | Capsid-specific restriction factor that prevents infection from non-host-adapted retroviruses. |
| TRPM6 | 3 | Essential ion channel and serine/threonine-protein kinase. |
| TSHR | - | Receptor for the thyroid-stimulating hormone (TSH) or thyrotropin. |
| TTC12 | - | Cytoplasmic protein that plays a role in the proper assembly of dynein arm complexes in motile cilia in both respiratory cells and sperm flagella. |
| UBASH3B | - | Interferes with CBL-mediated down-regulation and degradation of receptor-type tyrosine kinases. |
| UBN1 | - | Acts as a novel regulator of senescence. |
| VAMP4 | - | Involved in the pathway that functions to remove an inhibitor (probably synaptotagmin-4) of calcium-triggered exocytosis during the maturation of secretory granules. |
| VAV3 | - | Exchange factor for GTP-binding proteins RhoA, RhoG and, to a lesser extent, Rac1. Binds physically to the nucleotide-free states of those GTPases. |
| VLDLR-AS1 | - | Unknown |
| WAC | 1,S | Acts as a linker between gene transcription and histone H2B monoubiquitination at 'Lys-120' (H2BK120ub1). |
| WDPCP | - | Probable effector of the planar cell polarity signaling pathway which regulates the septin cytoskeleton in both ciliogenesis and collective cell movements. |
| WEE2-AS1 | - | Unknown |
| Z82190.2 | - | Unknown |
| ZBTB44-DT | - | Unknown |
| ZC3H3 | - | Required for the export of polyadenylated mRNAs from the nucleus. |
| ZFHX3 | - | Transcriptional regulator which can act as an activator or a repressor. |
| ZNF219 | - | Transcriptional regulator. Recognizes and binds 2 copies of the core DNA sequence motif 5'-GGGGG- 3'. |
| ZNF276 | - | May be involved in transcriptional regulation. |
| ZNF438 | - | Isoform 1 acts as a transcriptional repressor. |
| ZNF676 | - | May be involved in transcriptional regulation. |
